# Data on charge separation of bispecific and mispaired IgGs using native charge-variant mass spectrometry

**DOI:** 10.1016/j.dib.2020.105435

**Published:** 2020-03-16

**Authors:** Wilson Phung, Guanghui Han, Stéphanie G.I. Polderdijk, Michael Dillon, Whitney Shatz, Peter Liu, Bingchuan Wei, Pawankumar Suresh, David Fischer, Christoph Spiess, Aaron Bailey, Paul J. Carter, Jennie R. Lill, Wendy Sandoval

**Affiliations:** a Departments of Microchemistry, Proteomics and Lipidomics, Genentech, Inc., 1 DNA Way, South San Francisco, CA, USA; b Antibody Engineering, Genentech, Inc., 1 DNA Way, South San Francisco, CA, USA; c Protein Chemistry, Genentech, Inc., 1 DNA Way, South San Francisco, CA, USA; d Protein Analytical Chemistry, Genentech, Inc., 1 DNA Way, South San Francisco, CA, USA; e San Jose Mass Spectrometry Center, BGI Americas, San Jose, CA, USA

**Keywords:** Bispecific antibody, Cognate pairs, Single cell, Charge variant mass spectrometry, Ion exchange chromatography, Weak cation exchange, Native mass spectrometry, Molecular model

## Abstract

The data supplied in this work are related to the research article entitled “Characterization of Bispecific and Mispaired IgGs by Native Charge-Variant Mass Spectrometry” (Phung et al., 2019). This data article describes a powerful analytical platform using native weak cation exchange chromatography coupled to a high-resolution mass spectrometer, charge variant mass spectrometry (CV-MS), to characterize bispecific and mispaired antibody species. Elution order is investigated through analytical methods and molecular modeling in an effort to understand the intrinsic charge, size and shape differences of these molecules.

Specifications table

**Table:** 

Subject	Analytical chemistry
Specific subject area	Native mass spectrometry
Type of data	UV absorption chromatograms, intact mass spectra, molecular modeling figures, pH titration curve graphs
How data were acquired	Thermo Exactive Plus EMR Orbitrap mass spectrometer with Xcalibur and Protein Deconvolution software; Shimadzu FRC-10A fraction collector and LabSolutions software; BioLuminate (Schrödinger) Build Antibody software; Wyatt Technology Corporation ASTRA software
Data format	Analyzed data, raw data
Parameters for data collection	Various pH gradients were tested to obtain optimal chromatographic separation of bispecific and mispaired antibodies
Description of data collection	Mass spectrometric data obtained were raw data analyzed in Xcalibur and Protein Deconvolution software; antibody sequences were used for molecular modeling and generation of pH titration curves in BioLuminate (Schrödinger) Build Antibody software
Data source location	Genentech, Inc., Department of Microchemistry, Proteomics and Lipidomics, Department of Antibody Engineering, Department of Protein Chemistry, and Department of Protein Analytical Chemistry
Data accessibility	Data are presented with this article
Related research article	W. Phung, G. Han, S. Polderdijk, M. Dillon, W. Shatz, P. Liu, B. Wei, P. Suresh, D. Fischer, C. Spiess, A. Bailey, P. Carter, J. Lill, and W. Sandoval. Characterization of Bispecific and Mispaired IgGs by Native Charge-Variant Mass Spectrometry. International Journal of Mass Spectrometry, Volume 446, 2019, 116229, https://doi.org/10.1016/j.ijms.2019.116229

## Value of the data

• Charge variant mass spectrometry (CV-MS) was utilized to successfully separate the correctly paired and light chain-scrambled mispaired isobaric species which may occur during bispecific antibody production in single host cells.
• Scientific researchers in a variety of settings can utilize CV-MS to separate proteoforms based on only minor charge differences and directly analyze by mass spectrometry.
• By charge separation of correctly paired and mispaired species, downstream purification of BsIgGs are made possible as an alternative to the incorporation of chain pairing strategies.
• Molecular modeling revealed that minor surface exposed charge patches created microenvironments which were sufficient to resolve species, but not significant enough to alter the molecular isoelectric point.

## 1 Data

Our data demonstrates an integrated native ion exchange chromatography mass spectrometry based analytical method, CV-MS [1,2], to successfully separate correctly paired and mispaired IgG species which may arise during bispecific antibody production in single host cells [1].

**Fig. 1:**
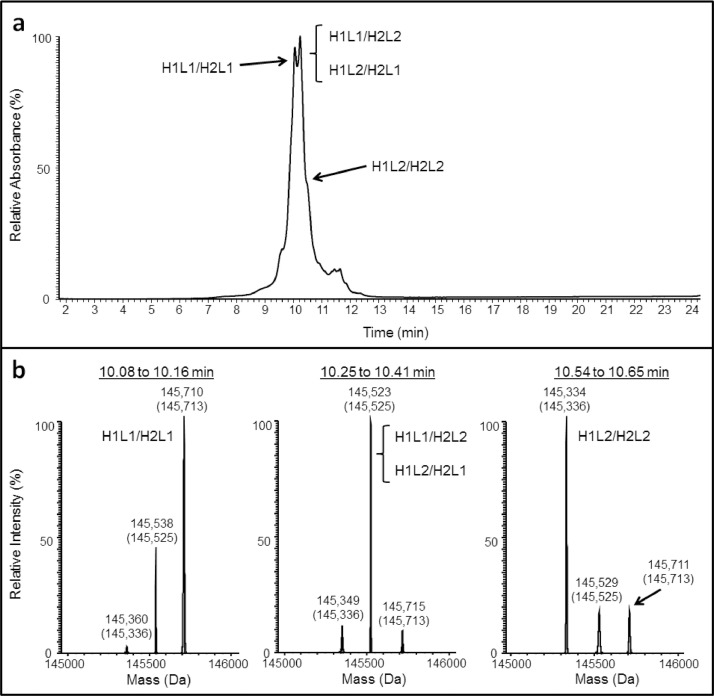
RPLC-MS analysis of anti-HER2/CD3 BsIgG on HALO RP column showed minor separation of IgG species. (a) UV absorption chromatogram (280 nm) (b) Deconvoluted mass spectrum. Theoretical masses are denoted in parentheses.

**Fig. 2:**
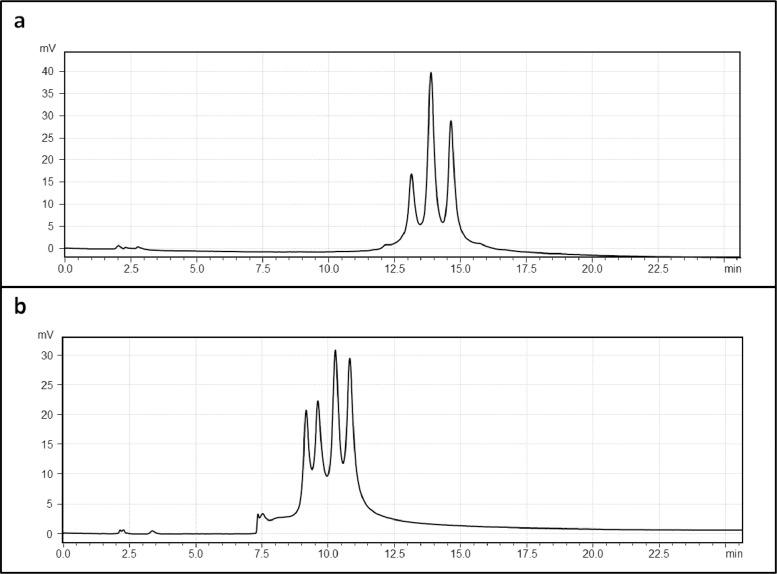
Ion exchange comparison of salt (a) and pH (b) gradients for separating anti-HER2/CD3 BsIgG.

**Fig. 3:**
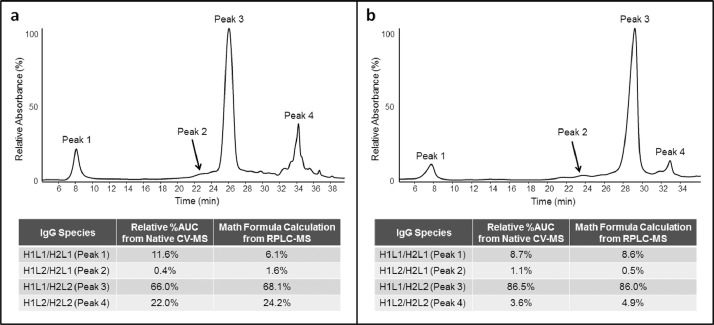
Native CV-MS analysis of anti-IL-13/IL-4 and anti-EGFR/MET with improved peak separation after pH gradient optimization. UV absorption chromatogram for (a) anti-IL-13/IL-4 and (b) anti-EGFR/MET.

Anti-HER2/CD3 BsIgG was analyzed by reversed phase HPLC using an organic solvent gradient (Fig. 1) and traditional ion exchange using a salt gradient (Fig. 2(a)). We were not able to resolve isobaric species with extensive method optimization. A commercially available pH buffer system (CX-1) was also used to confirm the separation of IgG species with pH gradient cation chromatography (Fig. 2(b)), but these buffers are incompatible with downstream mass spectral analysis. Charge variant-mass spectrometry (CV-MS) was the only method demonstrated to resolve BsIg scrambled species, and holds the additional benefit of mass spectral detection for species identification [1]. To demonstrate the separation of impurities in a variety of paired single cell half antibody IgG assemblies by CV-MS, anti-IL-13/IL-4 and anti-EGFR/MET were also analyzed after optimizing the pH gradient for each BsIgG (Fig. 3) and show complete separation of the main peak from the BsIg scramble.

**Fig. 4:**
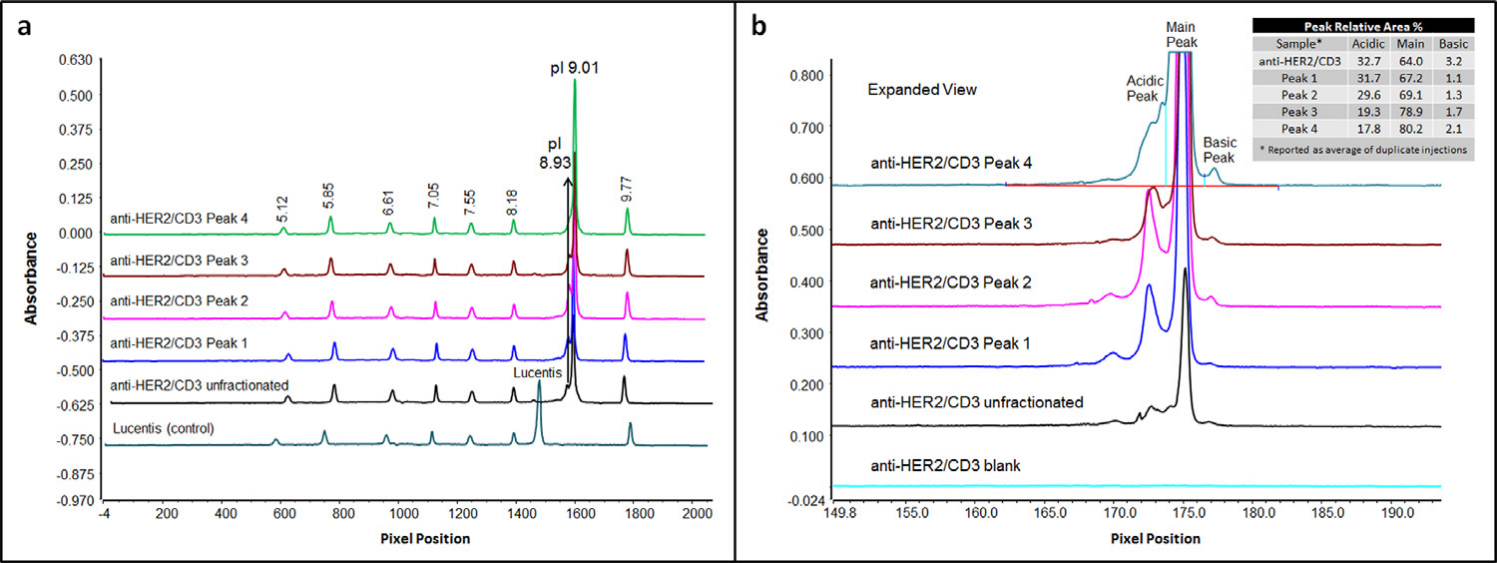
Isoelectric point determination of anti-HER2/CD3 peaks post-fractionation by iCE3 (a) or iCIEF (b).

**Fig. 5:**
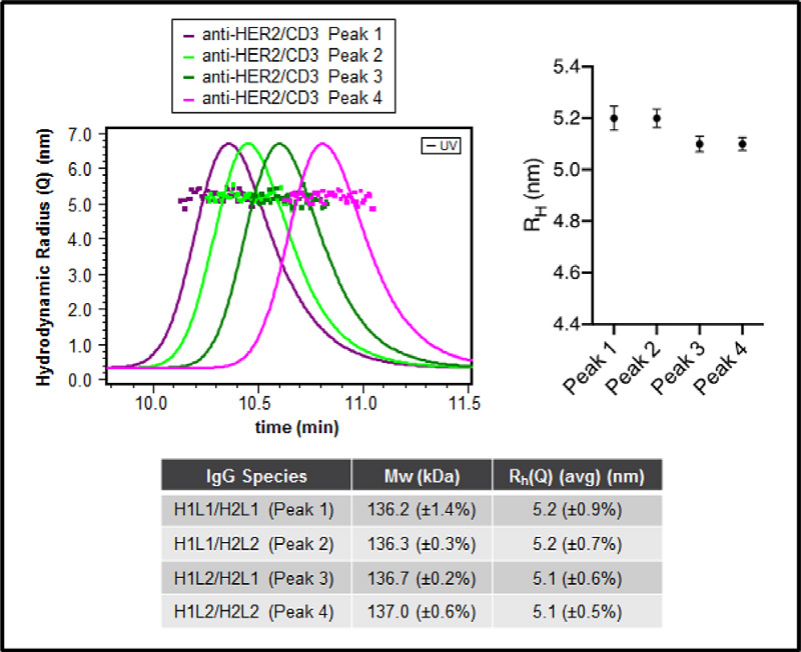
Hydrodynamic radius determination of anti-HER2/CD3 peaks post-fractionation.

**Fig. 6:**
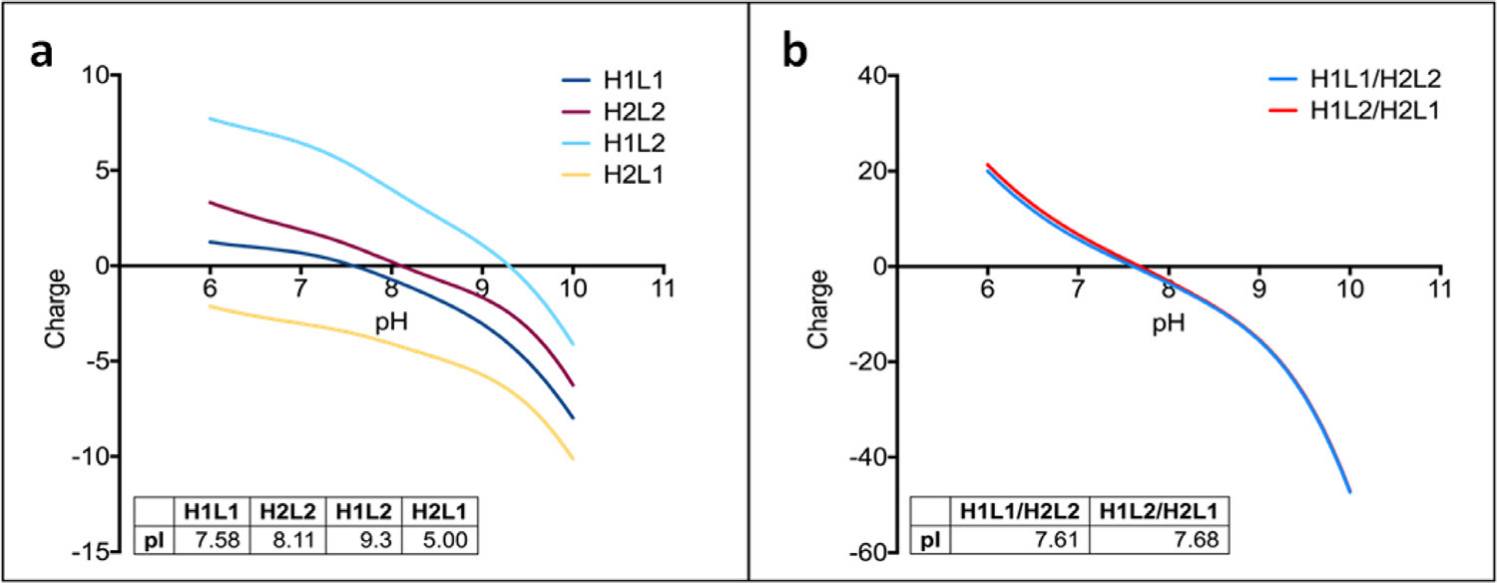
Model charge over the indicated pH gradient for anti-IL-13/anti-IL-4 Fv and BsIgG species. (a) Fv domains only (1 = anti-IL-13, 2 = anti-IL-4). (b) Full BsIgG models.

**Fig. 7:**
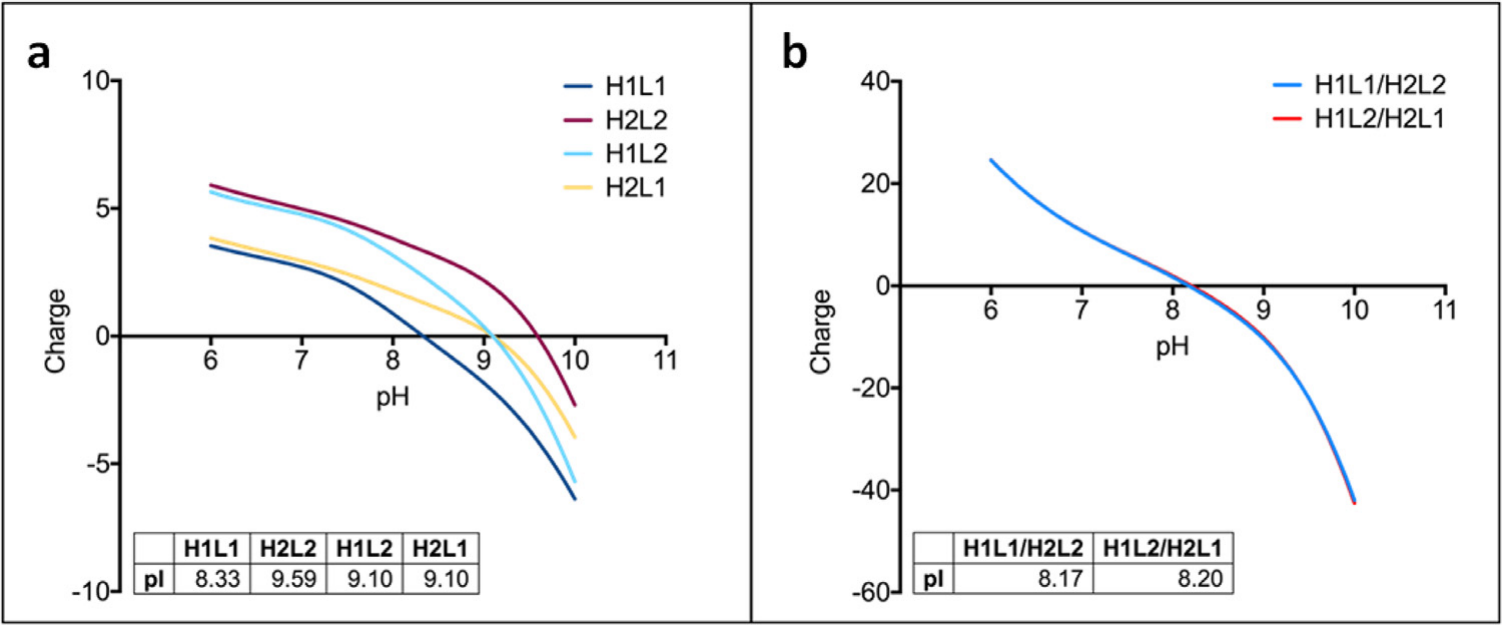
Model charge over the indicated pH gradient for anti-EGFR/anti-MET. (a) Fv domains only (1 = anti-EGFR, 2 = anti-MET). (b) Full BsIgG models.

**Fig. 8:**
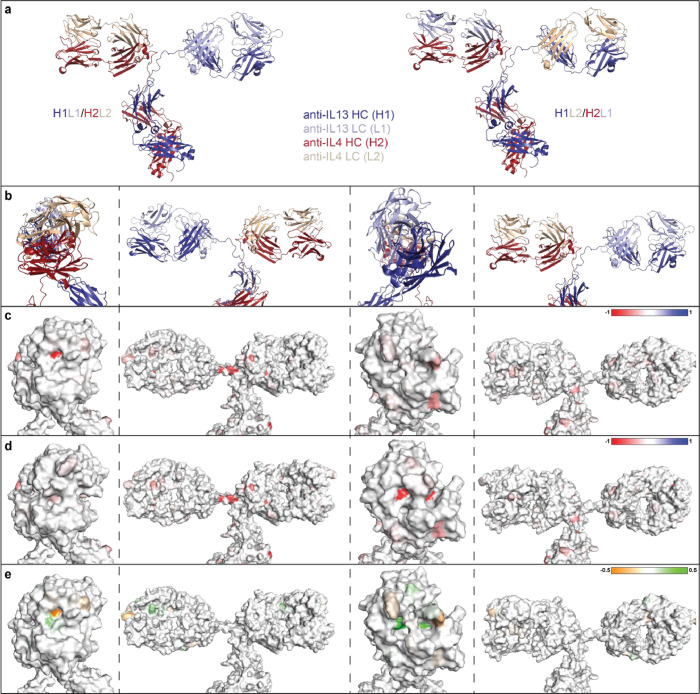
Localized charge changes in anti-IL-13/IL-4 BsIgG species over a pH range from 7 to 9. (a) Models of the bispecific that is either correctly paired or mispaired. Anti-IL-4 is shown in red (heavy chain) and wheat (light chain), anti-IL-13 is dark blue (heavy chain) and light blue (light chain). (b) Views of the correctly paired anti-IL-13/IL-4. For orientation the same views are used in (c–e). (c and d) Surface view of charge changes at pH 9 subtracted from pH 7 for each residue over pH gradient for (c) H1L1/H2L2 or (d) H1L2/H2L1. (e) Identical residue positions of maps (c) and (d) are subtracted for mapping onto the H1L1/H2L2 model. Areas of color change indicate regions with the largest difference charge change over the pH gradient between H1L1/H2L2 and H1L2/H2L1.

**Fig. 9:**
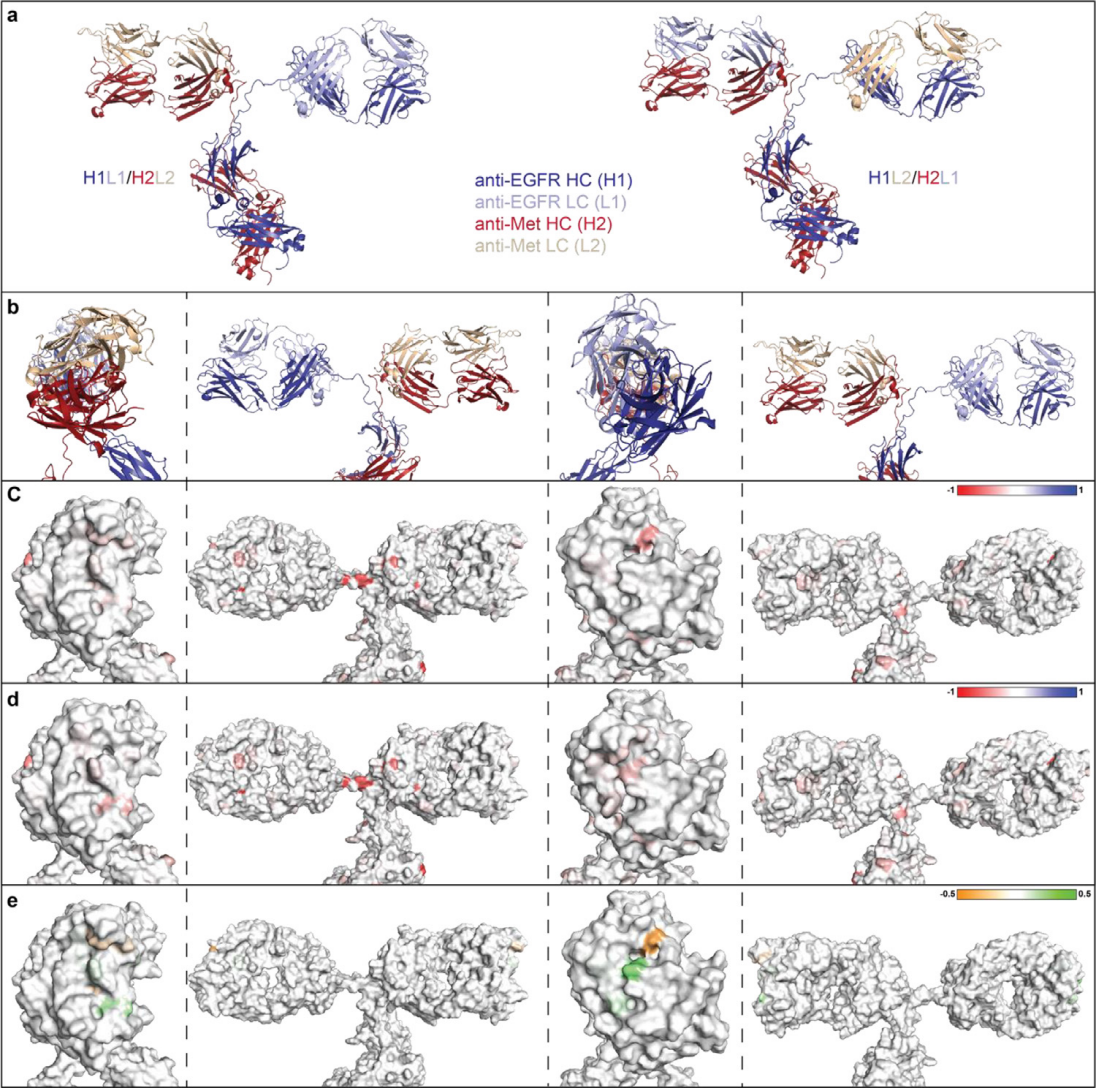
Localized charge changes in anti-EGFR/MET BsIgG species over a pH range from 7 to 9. (a) Correctly paired or mispaired anti-EGFR/MET BsIgGs. Anti-MET chains are red (heavy) and wheat (light), anti-EGFR chains are shown in dark blue (heavy) and light blue (light). (b) Correctly paired anti-EGFR/MET. (c and d) Surface view of charge changes at pH 9 subtracted from pH 7 for each residue over pH gradient for (c) H1L1/H2L2 or (d) H1L2/H2L1. (e) Subtraction between the maps in (c) and maps in (d) mapped onto the H1L1/H2L2 model.

To confirm the identity of isobaric species in the single cell anti-HER2/CD3 BsIg, each peak was fraction collected after charge separation. The isoelectric point was compared for each IgG species by iCIEF using iCE3 (Fig. 4). The hydrodynamic radius was also compared for each IgG species by SEC-MALS (Fig. 5). Lastly, molecular modeling with BioLuminate was used to generate protein titration curves of charge over pH for anti-HER2/CD3 in the research article [1], as well as two other molecules, anti-IL-13/IL-4 and anti-EGFR/MET (Fig. 6, Fig. 7). Molecular modeling was employed to localize charge changes for each IgG species over a pH range from 7 to 9 and the surface exposed charge patches were compared (Fig. 8, Fig. 9).

## 2 Experimental design, materials, and methods

Below is a brief description of the experimental methods used to acquire data in this paper. For a more detailed and thorough report, please refer to the related research article [1].

### 2.1 Native CV-MS

Bispecific antibodies were buffer exchanged into 50 mM ammonium acetate and separated on a ProPac WCX-10 column (Thermo Scientific) using gradients optimized for each individual BsIgG. The HPLC was coupled to a Thermo Exactive Plus EMR Orbitrap (Thermo Fisher Scientific). Acquired data was processed with Thermo Xcalibur Qual Brower and Thermo Protein Deconvolution 4.0.

### 2.2 Offline reversed phase separation and ion exchange

Separation of anti-HER2/CD3 BsIgG was evaluated on a HALO RP column and analyzed on a Thermo Exactive Plus EMR Orbitrap instrument for native CV-MS comparison. Anti-HER2/CD3 BsIgG was also buffer exchanged into 50 mM ammonium acetate, pH 7 and separated offline using a ProPac WCX-10 column on a Shimadzu FRC-10A instrument. Traditional IEC salt gradient and commercial pH gradient (CX-1 pH Gradient Buffer Kit, Thermo Scientific), which are incompatible with downstream mass spectral analysis, were used for offline separation and compared to native CV-MS.

### 2.3 Comparison of anti-HER2/CD3 bispecific and mispaired IgGs by iCIEF and SEC-MALS

Buffer exchanged anti-HER2/CD3 BsIgG was separated and fractionated offline using a ProPac WCX-10 column on a Shimadzu FRC-10A instrument. Fractionated anti-HER2/CD3 bispecific and mispaired IgGs were analyzed by iCIEF using iCE3 to determine differences in isoelectric point. Fractionated samples were also analyzed by SEC-MALS using quasi elastic light scattering (QELS) to determine differences in hydrodynamic radius. The Stokes-Einstein relationship was used to calculate R_H_ from measured diffusion coefficients.

### 2.4 Molecular modeling

Initial Fv models with correctly paired chains were built using the commands described in the main research article.

Templates used for the respective Fv models are given in the table below.

**Table:** 

Model	HC Fv template	LC Fv template	Framework template	HC loop templates	LC loop templates

4D5_Fv	6BAH_B	5EU7_C	1N8Z_A	H1: 3MLT H2: 5UEA H3: 1N8Z	L1: 5TD0 L2: 1DQQ L3: 5TDP
CD3_Fv	3EO9_H	2WUC_L	3EO9_L	H1: 6AQ7 H2: 5VF2 H3: 4EDX	L1: 5U4R L2: 3CMO L3: 1GHF
4D5HC_CD3LC_Fv	6BAH_B	2WUC_L	3BDY_L	H1: 3MLT H2: 5UEA H3: 1N8Z	L1: 5U4R L2: 3CMO L3: 1GHF
CD3HC_4D5LC_Fv	3EO9_H	5EU7_C	3EO9_L	H1: 6AQ7 H2: 5VF2 H3: 4EDX	L1: 5TD0 L2: 1DQQ L3: 5TDP
IL13_Fv	4I77_H	3INU_L	4I77_L	H1: 1FN4 H2: 2HWZ H3: 5EZJ	L1: 4I77 L2: 5EOC L3: 4HS6
IL4_Fv	5EU7_E	2WUC_L	4G7V_L	H1: 3BPC H2: 4LRI H3: 1IGT	L1: 5TKK L2: 2Q8B L3: 4LVE
IL13HC_IL4LC_Fv	4I77_H	2WUC_L	6APB_L	H1: 4I77 H2: 2HWZ H3: 4I77	L1: 5TKK L2: 2Q8B L3: 4LVE
IL4HC_IL13LC_Fv	5EU7_E	3INU_L	3INU_L	H1: 3BPC H2: 4LRI H3: 1IGT	L1: 4I77 L2: 5EOC L3: 4HS6
EGFR_Fv	3P0V_H	2WUC_L	3P0V_L	H1: 3FB5 H2: 3N9G H3: 4NYL	L1: 3SOB L2: 2R8S L3: 6APB
MET_Fv	4K3J_H	2WUC_L	4K3J_L	H1: 1NGZ H2: 2XQY H3: 4G7V	L1: 4K3J L2: 5IFA L3: 1ZA6
EGFRHC_METLC_Fv	3P0V_H	2WUC_L	3P0V_L	H1: 3FB5 H2: 3N9G H3: 4NYL	L1: 4K3J L2: 5IFA L3: 4LVE
METHC_EGFRLC_Fv	4K3J_H	2WUC_L	4K3J_L	H1: 1NGZ H2: 2XQY H3: 4G7V	L1: 3SOB L2: 2R8S L3: 6APB

Charge and pKa of the individual residues were calculated using the BioLuminate Protein Titration Curve tool (Schrödinger, Suites 2018-4) in 0.1 increments over a range of pH 6–10.

Models of the correctly paired and light chain-scrambled species were aligned in BioLuminate and renumbered. Alignment and numbering were checked and renumbered manually if required.

Difference in charge between pH 9 and pH 7 was calculated, as were the differences in change of charge per residue as described in the reference article.

## Conflict of Interest

WP, SP, MD, WS, PL, BW, DF, CS, PJC, JRL and WS are current employees of Genentech, Inc., which develops and markets drugs for profit. GH and PS were employees for Genentech at the time of these experiments. AOB is a former employee of Thermo Fisher Scientific, the company who manufactures and markets the chromatography and mass spectrometry instrumentation used.

## Appendix Supplementary materials

Image, application 1
